# Whatever after Next? Adaptive Predictions Based on Short- and Long-Term Memory in Visual Search

**DOI:** 10.3389/fpsyg.2012.00409

**Published:** 2012-10-18

**Authors:** Markus Conci, Martina Zellin, Hermann J. Müller

**Affiliations:** ^1^Department Psychologie, Ludwig-Maximilians-Universität MünchenMunich, Germany

Generating predictions for task-relevant goals is a fundamental requirement of human information processing, as it ensures adaptive success in our complex natural environment. Clark (in press) proposed a model of hierarchical predictive processing, in which perception, attention, and learning are unified within a coherent framework. In this view, incoming sensory signals are constantly matched with top-down expectations or predictions, with the aim of minimizing the prediction error to generate adaptive behavior. For example, in a natural environment such as a kitchen, search for a given target object (e.g., a pan) might be guided by a variety of predictive cues generated by previously acquired knowledge, such as the target’s typical appearance (e.g., its color, size, and shape as defined by a top-down implemented search template). In addition, predictions can also be derived from contextual factors, such as the most probable location of the target (e.g., on the stove), and its typical co-occurrence with other objects (e.g., pan and kettle; see Oliva and Torralba, 2007; Wolfe et al., 2011, for reviews).

Clark’s proposal essentially describes the human brain as a “prediction machine,” which is well suited to explain adaptation within the context of learning and memory function. Here, we would like to argue that the temporal scale of using and adjusting predictions offers a valuable additional perspective to hierarchical predictive processing. More specifically, predictions might be based on both short-term learning (the focus of Clark’s proposal) and on long-term memory associations. For example, in visual search, the recent history of target features and locations can be used to quickly adjust spatial attention to task-relevant objects in a scene, thus providing a prediction on the basis of recent experience (see, e.g., Kristjánsson and Campana, 2010, for a review of intertrial priming). Furthermore, predictive processing can also be derived from long-term learning. For instance, observers may extract the statistical co-occurrence of shapes in a rather automatic and rapid fashion and subsequently employ these probabilities of co-variation for object detection (e.g., Fiser and Aslin, 2001). Higher-order statistical scene regularities can also guide the deployment of attention. For example, consistent associations between a given target location and its surrounding spatial context are implicitly learned and guide spatial attention in visual search (“contextual cueing”; Chun and Jiang, 1998). These contextual associations are acquired after only a few repetitions, but show long-lasting persistence (Chun and Jiang, 2003). Taken together, evidence from visual statistical learning suggests that the brain is very efficient at detecting “suspicious coincidences” of object co-variation in the environment, which then shape predictions about upcoming events and guide behavior across both shorter and longer periods of time.

An important characteristic of predictive processing is the potential to adjust expectations through error minimization in a dynamic manner. For instance, top-down predictions should be adapted if they differ from bottom-up sensory signals, because task-relevant targets might change frequently. In fact, relevant target features can be adjusted from one instance to the next, with relatively transient switch costs after a change (Maljkovic and Nakayama, 1994; Found and Müller, 1996). Moreover, in some cases, two parallel and competing prediction models configure behavior, for example when perceiving ambiguous figures (e.g., a bistable duck-rabbit figure; see Brugger and Brugger, 1993) – leading to constant switches between equally likely perceptual interpretations. Thus, some evidence, which seems to relate in particular to short-term predictions, suggests that expectations are dynamically adjustable based on encountering only a few instances.

However, other studies show a limited amount of adaptive resources, in particular for longer-lasting predictions. For example, incidental learning of statistical regularities is usually not adapted rapidly: an attentional bias toward a predicted target location (i.e., the most likely target location) is not rapidly readjusted to change and may persist for long periods of time (Jiang et al., 2012). Moreover, learning of context-target associations is typically limited to a single target location; no further target locations are associated with a given invariant context (Zellin et al., 2011). In addition, the adaptation of learned contextual associations after a change of the target location is rather inflexible, resulting in the persistence of a misleading cue (Manginelli and Pollmann, 2009). In fact, adaptation only occurred for changes that were initially predictable (Conci et al., 2011; Conci and Müller, 2012). Taken together, these findings suggest that predictive processing is rather inflexible in dynamically adjusting to changing sensory signals in statistical learning.

In sum, we would like to suggest that predictive processing is involved in immediate and long-term learning, extending the framework proposed by Clark. In addition, prediction models may be differentiated according to restrictions on adaptive processes, with a larger degree of flexibility for predictions based on short-term as opposed to long-term learning. Short-lived predictions may require adaptive resources to dynamically account for frequent changes in the recent history. On the other hand, non-adaptive models might, in some instances, be more reliable and less demanding in terms of processing load based on the assumption (or “hyperprior”) that the environment is rather invariant. Thus, given that the world is typically relatively stable, unpredictable changes should barely affect predictions because they represent an exception rather than the norm. The degree of available error minimization may therefore vary for different predictions and can eventually result in inadequate behavioral adaptation.
